# Epidemiological characteristics of pulmonary tuberculosis in Anhui Province, Eastern China from 2013 to 2018

**DOI:** 10.1371/journal.pone.0237311

**Published:** 2020-08-06

**Authors:** Qing-Qing Zhu, Qian Wu, Ai-Min Wang, Fang-Jin Bao, Yong-Zhong Zhang, Jie Liu, Jun-Wei Yan, Xue-Hui Fang, Ling Li, Ze-Kun Zhang, Rong Wang, Xun-Di Bao, Song Yao, Hai-Feng Pan

**Affiliations:** 1 Anhui Provincial Tuberculosis Institute, Hefei, Anhui, China; 2 Department of Epidemiology and Biostatistics, School of Public Health, Anhui Medical University, Hefei, Anhui, China; The Chinese University of Hong Kong, HONG KONG

## Abstract

**Objective:**

Pulmonary tuberculosis (TB) is a severe infectious respiratory disease, the burden of which remains high in China. To provide scientific evidence for developing more targeted prevention and control strategies, this study aimed to determine the incidence trends and explore the epidemiological characteristics of pulmonary TB in Anhui Province, Eastern China between 2013 and 2018.

**Methods:**

The retrospective study analyzed information regarding pulmonary TB cases reported by the National Infectious Disease Reporting System and census data collected from the Anhui Provincial Bureau of Statistics.

**Results:**

Overall, 211,892 cases of TB patients were reported in Anhui Province, China between 2013 and 2018, with an average annual reported incidence rate of 57.7 per 100,000 persons. A significant decrease in the incidence rate of pulmonary TB (*p <* 0.001) was observed during the study period. Men had a higher incidence rate of pulmonary TB than women (*p* < 0.001). The highest annual average reported incidence rate was 204.2 per 100,000 persons in those aged 70–74 years. The number of farmers with pulmonary TB, i.e., 155,415, accounted for 73.4% of all cases. Moreover, the peak period of reported cases was from January to March. Four cities along the Yangtze River—Anqing, Tongling, Chizhou, and Wuhu—reported significantly higher incidence rates of pulmonary TB than other cities (*p* < 0.001).

**Conclusions:**

From 2013 to 2018, there was a significant decline in the incidence rate of pulmonary TB in Anhui Province, with peaks occurring from January to March. Prevention and control strategies targeting men, people aged 70–74 years, farmers, and the four cities along the Yangtze River should be strengthened.

## Introduction

Tuberculosis (TB) is an infectious disease caused by the bacillus *Mycobacterium tuberculosis (M*. *tuberculosis)*, which most commonly affects the lungs (pulmonary TB) and spreads when people with pulmonary TB expel the bacteria into the air [1, 2]. TB can lie dormant in individuals for decades and may be reactivated later, leading to widespread systemic symptoms [3]. This infectious disease is among the 10 leading causes of death worldwide, ranking above HIV/AIDS [4, 5]. In 2015, the World Health Organization (WHO) approved the ambitious post-2015 global “End TB Strategy” [6] with a goal of reducing TB incidence by 90% and TB deaths by 95% by 2035 [7]. To end the global TB epidemic, improved diagnostic tools and treatment as well as effective surveillance targeting the vulnerable populations are imperative.

Despite extensive control strategies and increased public awareness, the disease remains an enormous threat to global public health worldwide [8]. According to WHO reports, an estimated 10 million people were living with TB in 2017, with an incidence rate of 133.0 per 100,000 persons [9]. As the country with the second-highest TB burden worldwide, China has the third-highest number of cases [10]. According to statistics from the National Information System For Disease Control And Prevention (NISDCP), the reported incidence rate of TB in Anhui Province (55.8 per 100,000 persons) in 2017 ranked 28th among the 31 provinces (autonomous regions and municipalities). Moreover, the number of pulmonary TB cases reported in Anhui Province (34,561) accounted for 4.1% of new cases nationwide, ranking ninth in the country.

Anhui Province, an important agricultural production base in the east of China, has a large proportion of farmers. Although the incidence rate of pulmonary TB in Anhui Province from 2013 to 2018 was roughly equal to the national average, the number of reported cases ranked 10^th^ in China, according to data from the NISDCP. We conducted this six-year retrospective study to provide a scientific basis for a deeper understanding of epidemiological trends of pulmonary TB in Anhui Province and to develop more targeted prevention and control strategies.

## Materials and methods

### Diagnostic criteria

According to the diagnostic criteria for pulmonary TB issued by the National Health Commission of the People’s Republic of China (WS288–2008), pulmonary TB patients should meet the following criteria: 1) suggested TB symptoms (e.g., cough for more than 2 weeks or hemoptysis), 2) sputum specimens smear-positive for acid-fast bacilli, 3) negative sputum smears but with pulmonary lesions on chest radiographs [11].

### Data collection

Data regarding the reported TB cases in Anhui Province from January 2013 to December 2018 were obtained from the National Infectious Disease Reporting System (NIDRS), the subsystem of the NISDCP. Population data were collected from the Anhui Provincial Bureau of Statistics. According to the Chinese law of Infectious Disease Control and Prevention, all suggested and confirmed cases of pulmonary TB are required to be reported through the NIDRS within 24 hours. The reported information includes patient name, sex, age, occupation, residential address, date of onset and diagnosis as well as the location of the designated medical institution.

### Statistical analysis

The incidence rates of pulmonary TB (per 100,000 persons) were calculated by dividing the number of cases reported annually by the NIDRS by the annual population collected from the Anhui Provincial Bureau. Census subjects referred to the local residents who had lived continuously for more than 6 months, excluding mobile immigrants. The data were collected by using Excel 2007. To assess the trends in incidence rates, we applied Joinpoint regression analysis using Joinpoint Regression Program version 4.7.0.0 (https://surveillance.cancer.gov/joinpoint/). Chi-square tests for trends were conducted using IBM SPSS Statistics for Windows, version 23.0. MapInfo software, version 15.0 was used to map the geographic distributions. The significance tests were two-sided, with *p* values ≤ 0.05 considered statistically significant.

### Ethical approval

Ethics approval for this study was obtained from the Medical Ethics Committee of Anhui Provincial Tuberculosis Institute (FK2019-001). The data from the NIDRS were aggregated secondary data without any personal information; thus, informed consent was not needed.

## Results

### Reported incidence rate trends

**Fig 1:**
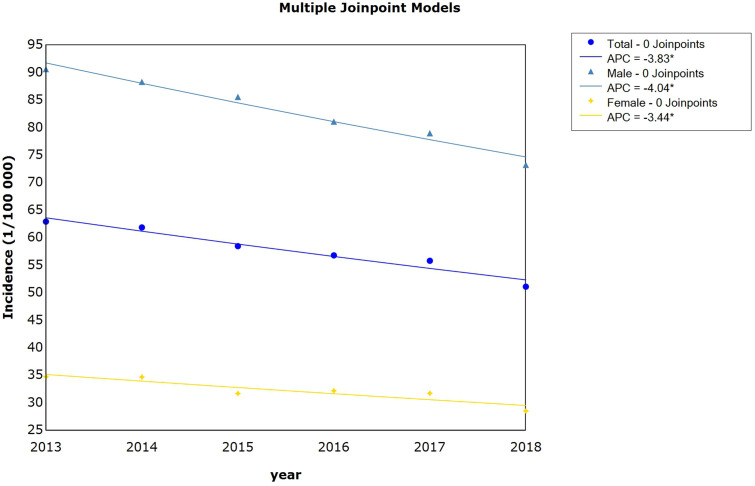
Trends in pulmonary tuberculosis incidence rates by sex in Anhui province, Eastern China from 2013 to 2018, as determined using the join-point regression analysis. APC, average percent change.

Between 2013 and 2018, a total of 211,892 cases of pulmonary TB were reported in Anhui Province, China, with an average annual reported incidence rate of 57.7 per 100,000 persons. The reported incidence rates decreased significantly from 62.9 cases per 100,000 persons in 2013 to 51.1 cases per 100,000 persons in 2018, with an average percent change (APC) of 3.8% (95% confidence interval [CI]: -5.0, -2.6; *p* < 0.05) (Fig 1).

### Sex distribution of TB patients

Among the pulmonary TB cases reported in Anhui Province from 2013 to 2018, 153,303 were that of men and 58,589 that of women, corresponding to a sex ratio of 2.62:1. The average annual reported incidence rate for men (82.85 per 100,000 persons) was significantly higher than that for women (32.21 per 100,000 persons) (*χ*^*2*^ = 40774.1, *p <* 0.001). From 2013 to 2018, significant decreases in the incidence rates of pulmonary TB were observed in both men (APC = -4.0, 95% CI: -5.0, -3.1; *p* < 0.05) and women (APC = -3.4, 95% CI: -5.6, -1.2; *p* < 0.05) (Fig 1).

### Age distributions of TB patients

Significant differences in pulmonary TB incidence rates were observed among different age groups. The highest annual average reported incidence rate was 204.2 per 100,000 persons in those aged 70–74 years. Moreover, the annual average reported incidence rate was the highest in men aged 80–84 years (319.4 per 100,000 persons) and in women aged 70–74 years (96.1 per 100,000 persons). From 2013 to 2018, the incidence rates of all age groups decreased significantly except for the 5–9, 10–14, 25–29, 45–49, and 50–54 years age groups (*p* < 0.001) (Table 2).

### Occupation distributions of TB patients

**Fig 2:**
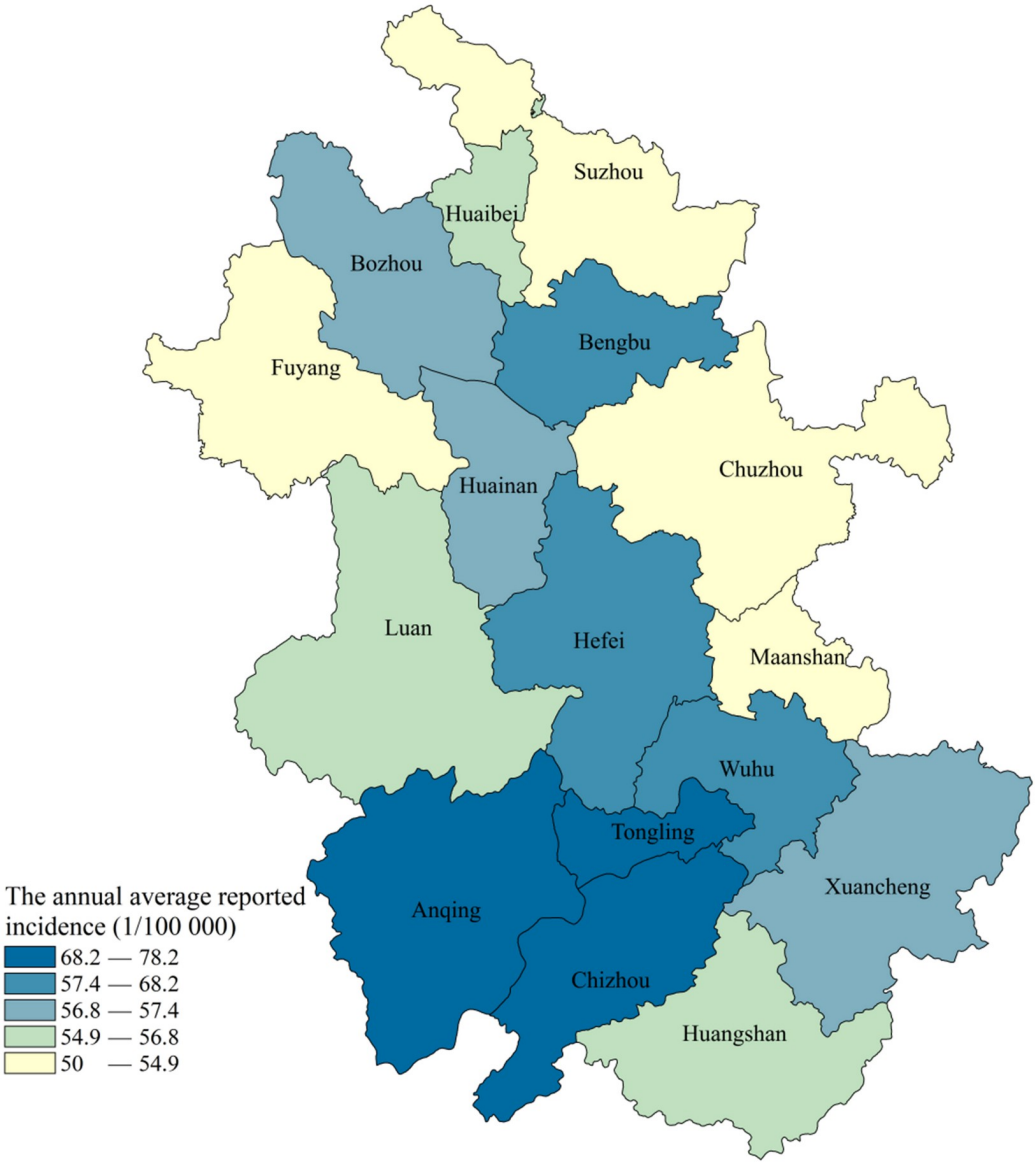
Geographic distributions of the average annual incidence of pulmonary tuberculosis in Anhui province, Eastern China, 2013–2018.

An analysis of the occupations of pulmonary TB patients reported in Anhui Province revealed 155,415 cases of farmers with pulmonary TB, accounting for 73.4% of the total number of pulmonary TB cases in this province. Domestic workers or unemployed people, retired people, and students accounted for 8.0%, 4.5%, and 3.8% of cases, respectively (Fig 2).

### Monthly distributions of TB patients

Between 2013 and 2018, the total monthly numbers of reported pulmonary TB cases showed a downward trend. The peak period for reported cases was from January to March, among which the number of reported cases in March was the highest throughout the years, with a total of 20,738 pulmonary TB cases reported (Table 1).

**Table 1:** Patient occupations and monthly distributions of reported pulmonary tuberculosis cases in Anhui province, Eastern China, 2013–2018.

Characteristics	Number (%)					
	2013	2014	2015	2016	2017	2018
**Occupation**						
**Kindergarten children**	1 (0.00)	2 (0.01)	3 (0.01)	2 (0.01)	2 (0.01)	2 (0.01)
**Scattered children**	23 (0.06)	18 (0.05)	34 (0.10)	26 (0.07)	21 (0.06)	12 (0.04)
**Students**	1434 (3.81)	1362 (3.65)	1299 (3.65)	1226 (3.52)	1346 (3.89)	1424 (4.46)
**Teachers**	158 (0.42)	121 (0.32)	127 (0.36)	101 (0.29)	108 (0.31)	136 (0.43)
**Nannies**	0 (0.00)	1 (0.00)	2 (0.01)	3 (0.01)	3 (0.01)	2 (0.01)
**Catering staff**	66 (0.18)	64 (0.17)	53 (0.15)	55 (0.16)	48 (0.14)	59 (0.18)
**Service personnel**	25 (0.07)	14 (0.04)	14 (0.04)	21 (0.06)	15 (0.04)	10 (0.03)
**Merchants**	704 (1.87)	625 (1.68)	876 (2.46)	870 (2.49)	964 (2.79)	859 (2.69)
**Medical staffs**	102 (0.27)	96 (0.26)	98 (0.28)	96 (0.28)	107 (0.31)	124 (0.39)
**Laborers**	1241 (3.29)	937 (2.51)	871 (2.45)	883 (2.53)	882 (2.55)	757 (2.37)
**Migrant workers**	546 (1.45)	171 (0.46)	147 (0.41)	171 (0.49)	130 (0.38)	123 (0.38)
**Farmers**	27963 (74.24)	27965 (74.99)	25958 (73.02)	25500 (73.11)	25044 (72.46)	22985 (71.94)
**Herders**	40 (0.11)	33 (0.09)	38 (0.11)	29 (0.08)	34 (0.10)	24 (0.08)
**Fishermen**	25 (0.07)	31 (0.08)	30 (0.08)	16 (0.05)	15 (0.04)	12 (0.04)
**Sailors and drivers**	37 (0.10)	30 (0.08)	26 (0.07)	19 (0.05)	20 (0.06)	18 (0.06)
**Cadres**	211 (0.56)	203 (0.54)	208 (0.59)	219 (0.63)	227 (0.66)	240 (0.75)
**Retired staffs**	1417 (3.76)	1559 (4.18)	1633 (4.59)	1543 (4.42)	1706 (4.94)	1672 (5.23)
**Unemployed people**	2412 (6.40)	3019 (8.10)	2954 (8.31)	3071 (8.80)	2896 (8.38)	2634 (8.24)
**Others**	554 (1.47)	513 (1.38)	663 (1.86)	762 (2.18)	710 (2.05)	650 (2.03)
**Unknown**	705 (1.87)	526 (1.41)	516 (1.45)	265 (0.76)	283 (0.82)	206 (0.64)
**Month**						
**January**	3388 (9.00)	3574 (9.58)	3482 (9.79)	3152 (9.04)	3182 (9.21)	3115 (9.75)
**February**	3325 (8.83)	3414 (9.16)	3097 (8.71)	3171 (9.09)	3176 (9.19)	2867 (8.97)
**March**	3643 (9.67)	3721 (9.98)	3577 (10.06)	3409 (9.77)	3134 (9.07)	3254 (10.18)
**April**	3648 (9.69)	3306 (8.87)	3143 (8.84)	2982 (8.55)	2921 (8.45)	2801 (8.77)
**May**	3297 (8.75)	3360 (9.01)	3173 (8.93)	3073 (8.81)	3041 (8.80)	2770 (8.67)
**June**	3164 (8.40)	3210 (8.61)	2924 (8.23)	2810 (8.06)	2920 (8.45)	2549 (7.98)
**July**	2903 (7.71)	3050 (8.18)	3167 (8.91)	2669 (7.65)	2708 (7.84)	2445 (7.65)
**August**	2929 (7.78)	2927 (7.85)	3003 (8.45)	2944 (8.44)	2908 (8.41)	2517 (7.88)
**September**	2836 (7.53)	2842 (7.62)	2614 (7.35)	2680 (7.68)	2692 (7.79)	2386 (7.47)
**October**	3008 (7.99)	2784 (7.47)	2637 (7.42)	2697 (7.73)	2732 (7.90)	2511 (7.86)
**November**	2724 (7.23)	2420 (6.49)	2314 (6.51)	2647 (7.59)	2607 (7.54)	2640 (8.26)
**December**	2799 (7.43)	2682 (7.19)	2419 (6.80)	2644 (7.58)	2540 (7.35)	2094 (6.55)

### Geographic distributions of TB patients

The average annual reported incidence rates of pulmonary TB (71.75 per 100,000 persons) in Anqing, Tongling, Chizhou, and Wuhu were significantly higher than those in the other 12 cities (54.58 per 100,000 persons) (*χ*^*2*^ = 2822.42, *p* < 0.001). Among the 16 cities in Anhui Province, the highest average annual reported incidence rates were in Anqing, Tongling, and Chizhou (Fig 2). In terms of geographic location, these four cities are distributed along the Yangtze River. From 2013 to 2018, the incidence rates in Hefei, Wuhu, Maanshan, Huaibei, Tongling, Anqing, Huangshan, Chuzhou, Fuyang, Suzhou, Luan, Bozhou, and Xuancheng showed significant downward trends (*p* < 0.001). However, the incidence rates in Chizhou increased significantly from 2013 to 2018 (*p* < 0.001) (Table 2).

**Table 2:** Trends in pulmonary tuberculosis incidence rates by age and region in Anhui province, Eastern China, 2013–2018.

Characteristics	2013	2014	2015	2016	2017	2018	*χ*^*2*^_*trend*_	p-value*
**Age (years)**								
** 0-**	0.82	0.43	0.60	0.49	0.42	0.31	7.95	0.005
** 5-**	0.45	0.40	0.40	0.59	0.24	0.31	1.48	0.223
** 10-**	3.37	2.95	3.25	3.53	4.16	3.46	2.09	0.148
** 15-**	40.28	38.51	36.22	36.03	33.33	27.02	116.41	<0.001
** 20-**	66.02	60.96	53.04	52.13	45.97	46.56	287.31	<0.001
** 25-**	63.16	66.39	68.57	68.45	68.16	60.90	0.41	0.524
** 30-**	42.82	42.06	40.92	41.36	41.01	39.20	5.52	0.019
** 35-**	35.60	34.02	31.81	30.23	30.45	26.23	72.55	<0.001
** 40-**	42.91	39.73	35.78	34.78	28.75	22.93	463.92	<0.001
** 45-**	39.69	41.77	38.38	36.96	36.18	42.96	0.83	0.362
** 50-**	116.76	141.47	131.55	141.33	142.48	115.98	0.17	0.685
** 55-**	78.84	65.18	58.68	54.70	52.42	57.61	191.89	<0.001
** 60-**	131.96	130.00	120.66	122.14	113.49	92.10	232.90	<0.001
** 65-**	165.31	162.21	153.57	131.09	145.51	132.89	126.99	<0.001
** 70-**	251.50	243.78	219.15	177.92	196.55	157.92	498.53	<0.001
** 75-**	224.53	215.84	197.38	182.61	196.10	155.00	183.31	<0.001
** 80-**	210.67	224.72	205.71	163.95	181.13	151.00	141.37	<0.001
** 85-**	125.98	134.59	130.50	107.99	128.26	113.71	6.00	0.014
**Region**								
** Hefei**	64.87	64.12	58.42	54.48	51.44	51.74	221.63	<0.001
** Wuhu**	64.85	67.09	65.48	61.63	67.17	59.23	8.38	0.004
** Bengbu**	59.98	57.65	53.99	53.54	66.77	54.49	0.01	0.905
** Huainan**	56.28	57.59	55.97	56.60	61.02	55.69	0.39	0.531
** Maanshan**	59.12	55.79	53.44	50.21	53.69	50.95	15.15	<0.001
** Huaibei**	56.72	56.33	53.26	58.33	52.14	52.87	4.07	0.044
** Tongling**	87.10	97.22	82.21	72.97	67.72	55.72	141.91	<0.001
** Anqing**	89.40	88.72	85.01	83.70	62.14	55.78	548.55	<0.001
** Huangshan**	60.83	61.51	53.69	53.21	53.88	51.30	17.64	<0.001
** Chuzhou**	60.64	56.09	55.93	50.14	49.75	46.03	103.15	<0.001
** Fuyang**	53.64	53.56	53.36	50.44	47.64	42.42	133.89	<0.001
** Suzhou**	54.24	54.20	49.95	50.88	46.61	46.28	59.36	<0.001
** Luan**	64.28	60.32	56.01	49.80	53.54	48.06	156.26	<0.001
** Bozhou**	61.38	58.43	55.09	56.11	59.68	50.47	31.37	<0.001
** Chizhou**	58.32	62.34	57.21	74.79	83.16	73.23	71.72	<0.001
** Xuancheng**	64.11	60.31	54.92	55.79	54.56	53.79	29.63	<0.001

*p-value: chi-square tests for trend

## Discussion

Despite being an ancient disease that has affected humankind for centuries, TB remains a major public health problem worldwide. The reported incidence rate of infectious diseases reflects the impact of diseases on people’s health as well as the distributions of these diseases. Additionally, the rate also reflects the reporting efficiencies of medical and health institutions. In the absence of factors such as under-reporting, a decline in reported incidence rate can be used to evaluate the rationality and effectiveness of current policies on the prevention and control of infectious diseases [12]. However, the progress in TB control has been slow in recent years, especially in low- and middle-income countries, owing to gaps in the coverage of TB programs and risk factors for TB transmission and progression [13]. Hence, there is an urgent need to explore the trajectory of the pulmonary TB epidemic and further evaluate whether China’s TB control and prevention plan can achieve the WHO goals on schedule.

This retrospective study analyzed the epidemiological distribution characteristics and temporal trends of pulmonary TB from 2013 to 2018 using data provided by the NIDRS. We found that the reported incidence rate dropped from 62.9 cases per 100,000 persons in 2013 to 51.1 cases per 100,000 persons in 2018, with an average annual decline rate of 4.1%. From 2013 to 2018, the incidence rate of pulmonary TB in Anhui Province was lower than the WHO’s estimate for China in the same year [9, 10, 14–17]. These improvements have primarily been attributed to the comprehensive prevention and control measures of *“12th Five-Year” and “13th Five-Year” National Tuberculosis Prevention and Control Plans* implemented by the General Office of the State Council of China, as well as the construction of new service and medical security systems [18].

The male/female sex ratio of pulmonary TB patients in Anhui Province reached 2.6, which was higher than that reported in Hebei Province, China [19]. We also found a significantly higher average annual reported incidence rate of pulmonary TB in men than in women. Differences in reported incidence rates between men and women may result from help-seeking behavior, stigma, socio-economic determinants/barriers, misdiagnosis (such as missed TB during pregnancy), etc. Men have a higher consumption of alcohol and cigarettes, both of which are risk factors for developing active TB [20–22]. Furthermore, a significant proportion of pulmonary TB cases was found in people aged 70–74 years, which was consistent with the findings from a cross-sectional study conducted in rural western China [23]. The host immune system plays a leading role in the containment and cure of *M*. *tuberculosis* infection [24]. However, T cell-mediated immunity undergoes an age-related decline in terms of its ability to respond to mycobacterial infection [25]. The aging of the population poses increasing challenges to TB control as China is considering its post-2015 End TB Strategy, which is not a new vertical program but rather a horizontal effort to comprehensively strengthen health care and expand coverage [26]. In this study, the proportions of pulmonary TB cases among farmers, domestic workers, and unemployed people were higher than those in other occupations, a finding consistent with national reports on the TB epidemic situation [27, 28]. Pulmonary TB transmission and progression are also driven by social factors. As Anhui Province is a major agricultural province with a large rural population base, poor living conditions, considerable economic burden, low levels of education, and malnutrition may account for the high incidence of pulmonary TB among peasants [29, 30].

Existing evidence has suggested periodic and seasonal features in the occurrence of pulmonary TB in China, with higher frequencies in winter and spring, hinting that the risk of pulmonary TB transmission appears to be the greatest during this period [31]. However, the mechanisms underlying the seasonal variation of pulmonary TB are complex and multi-factorial. In this study, we found that the incidence rate of pulmonary TB in Anhui Province was the highest from January to March. The peak months were earlier than those reported in other countries [32–34]. One possible explanation for seasonal variations of pulmonary TB is Vitamin D deficiency due to reduced sunlight exposure in winter, leading to immunosuppression and subsequent reactivation of latent TB infection [32, 35]. Moreover, indoor activities are more common in winter than in warm climates, which increases the probability of the exposure of healthy people to tubercle bacilli expelled from infected people [36, 37]. In particular, during the Spring Festival, most migrant workers return to their hometowns, thereby increasing population mobility and accelerating the transmission of tubercle bacilli [38, 39].

The incidence rates of pulmonary TB are reportedly higher in Anqing, Tongling, Chizhou, and Wuhu. As these areas are located in the Yangtze River Economic Belt of Anhui Province, their economic statuses are superior to those other mountainous and plain areas and the medical resources are relatively abundant, which is conducive to the early detection of pulmonary TB. As that low-income residents are more likely to delay seeking medical care in some densely populated rural areas, an increased focus on the equitable allocation of pulmonary TB-related resources and improved targeting of vulnerable groups may yield more timely diagnosis and treatment [40, 41].

This study has several limitations. First, this was a retrospective study based on programmatic data extracted from the NIDRS. The retrospective study design limited us in exploring the potential risk factors for the incidence rates. In addition, the possibility that the incidence was underestimated cannot be ruled out. Second, detailed information on the included pulmonary TB patients, such as nutritional status, living conditions, education level, smear results, and co-morbidities was not available from the medical records.

## Conclusions

From 2013 to 2018, the reported incidence rate of pulmonary TB in Anhui Province decreased significantly. Moreover, the incidence of pulmonary TB was higher among men and those aged 70–74 years. Among the new cases of pulmonary TB, farmers accounted for the largest proportion. The peak period of reported cases was from January to March. Four cities along the Yangtze River—Anqing, Tongling, Chizhou, and Wuhu—reported higher incidences of pulmonary TB than those in other areas. The results of this study may provide a scientific basis for public health decision-making and the promotion of effective measures for disease control.
